# Multiple aseptic splenic abscesses in a 15 year old patient

**DOI:** 10.1186/1471-230X-14-20

**Published:** 2014-02-06

**Authors:** Alexander J Jordan, Klaus-Peter Becker, Metin Sertemir, K Wolfgang Neff, Rüdiger Adam, Horst Schroten, Tobias Tenenbaum

**Affiliations:** 1 Paediatric Infectious Diseases, Department of Paediatrics, Medical Faculty Mannheim, Heidelberg University, Theodor-Kutzer-Ufer 1-3, 68167 Mannheim, Germany; 2 Institute for Medical Microbiology and Hygiene, Medical Faculty Mannheim, Heidelberg University, Mannheim, Germany; 3 Institute of Clinical Radiology and Nuclear Medicine, Medical Faculty Mannheim, Heidelberg University, Mannheim, Germany

**Keywords:** Abscess, Aseptic, Anaerobic bacteria, Metronidazole, Inflammatory bowel disease

## Abstract

**Background:**

Splenic abscesses in children are rare. In recent years aseptic abscesses have been recognized as a new disease entity, especially in adults.

**Case presentation:**

We present a rare case of a 15 year old girl with aseptic abscesses, in whom antibiotic therapy comprising metronidazole and meropenem was partly beneficial in improving the patient’s clinical condition and inflammatory parameters. Eventually corticosteroid therapy led to complete and long lasting resolution of symptoms. Further diagnostic work-up revealed autoimmune thyroiditis, but no signs of inflammatory bowel disease.

**Conclusion:**

Aseptic splenic abscesses should always prompt clinicians to initiate further diagnostics to determine a potential underlying condition and a regular follow-up. Anaerobic bacteria may play a role in the pathogenesis of the disease and besides corticosteroid treatment antibiotics covering anaerobes may be beneficial.

## Background

Splenic abscesses represent a rare disease entity which is even rarer in the paediatric age group. In most cases, these abscesses are caused by an infectious agent like *Staphylococcus aureus*, streptococcus spp., *Klebsiella pneumoniae*, *Escherichia coli*, *Mycobacterium tuberculosis*, or Salmonella spp. Risk factors constitute infections of the abdominal cavity, endocarditis, and immunosupression [1,2]. Malignant splenic lesions can also mimic abscesses if they are of the hypoechoic texture in ultrasound examination [3].

## Case presentation

**Figure 1:**
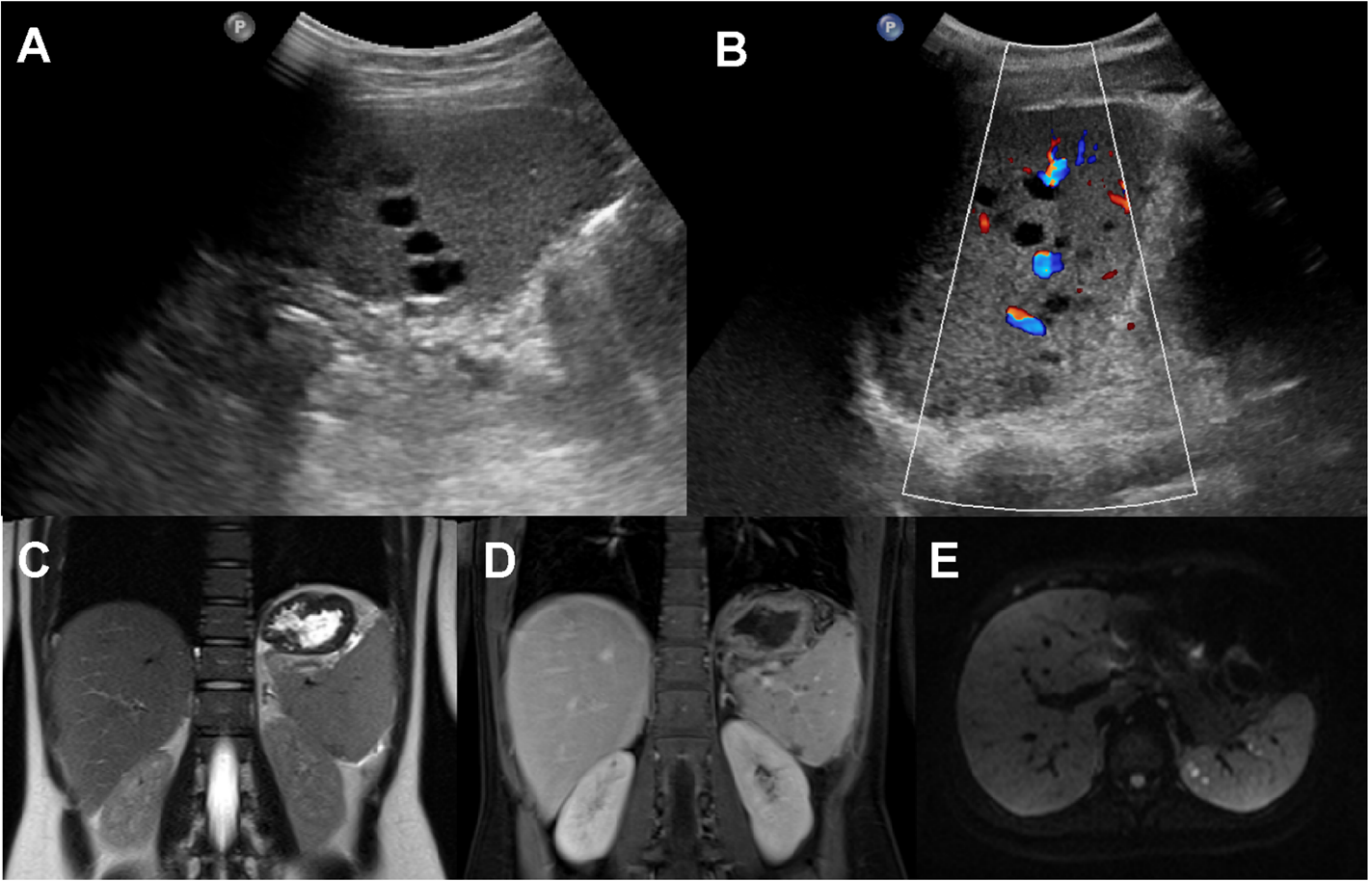
**Initial imaging of the abdomen. A**, **B**. Ultrasound of the spleen showing multiple an-/hypoechogenic masses without perfusion in the duplex sonography. **C-E**. MRI of the abdomen. **C**, the coronal T2w image demonstrates slightly hypo-/isointense areas in the spleen. **D**, the coronal postcontrast T1w image demonstrates hypointense lesions in the spleen, without contrast enhancement. **E**, the lesions show restricted diffusion on the transverse DW image (b 800 sec/mm^2^).

We report on a 15 year old adolescent girl that presented with a 10 day history of fever, aphthous putrid ulcers on the back of her throat and left upper quadrant abdominal pain radiating to the ipsilateral shoulder. Laboratory investigations revealed: white blood cell count (WBC) 14.8 × 10^9^/L (78.4% neutrophils, 12.7% lymphocytes), erythrocyte sedimentation rate (ESR) 56 mm/hour and C-reactive protein (CRP) 101 mg/L. Abdominal ultrasound showed multiple an-/hypoechoic lesions in the spleen with lack of perfusion in duplex sonography (Figure 1A, B). In abdominal magnetic resonance imaging (MRI) the splenic lesions showed a hypointense signal on the T1- and T2-weighted images without contrast enhancement; however, there was a diffusion restriction on the diffusion weighted (DW) images (Figure 1C-E). There was also localized contrast enhancement and restricted diffusion of the duodenal wall. The following endoscopic examination could not demonstrate any abnormalities within the duodenum. Histologic examination revealed mild chronic gastritis and duodenitis. An ultrasound study of the thyroid showed an inhomogeneous parenchyma with hypoechoic areas; thyroid function test showed normal peripheral hormone levels, but thyroid peroxidase-autoantibodies were elevated thus being suggestive for autoimmune thyreoiditis.

Serologic studies for acute infection with Epstein-Barr virus, cytomegalovirus, Herpes simplex-virus, Human immunodeficiency virus, *Mycoplasma pneumoniae*, *Yersinia enterica/pseudotuberculosis*, *Treponema pallidum*, *Coxiella burnetii*, Brucella spp., *Bartonella henselae*, *Franciscella tularensis*, and Echinococcus, as well as blood cultures were negative. A throat swab from the aphthous lesions showed normal resident flora. Urine analysis, chest X-ray and echocardiography as well as Mantoux test and interferon-gamma release assay were negative.

**Figure 2:**
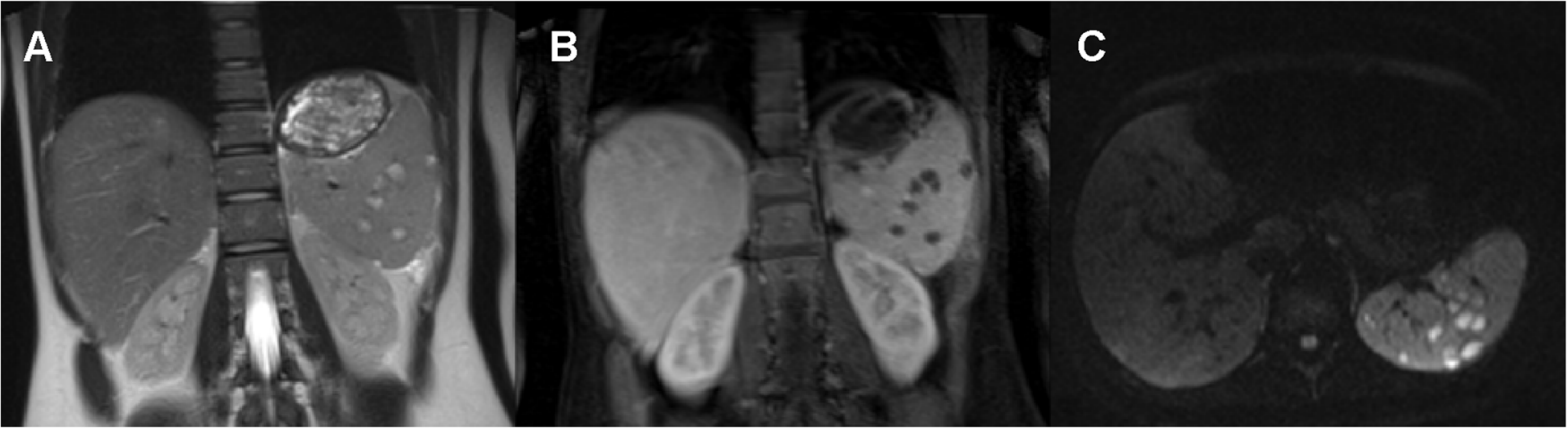
**Follow up MRI of the abdomen (24 days after admission). A**, the coronal T2w image shows hyperintense lesions with hypointense rim in the spleen. **B** and **C**, the coronal postcontrast T1w image and transverse DW image (b 800 sec/mm^2^), show an increased number and size of the lesions.

On admission the patient was started on ceftriaxone and clindamycine leading to no clinical improvement except cessation of fever but CRP even rose (129 mg/L). Therefore, antibiotics were switched to meropenem and metronidazole after 4 days. Under this regimen she physically improved, the pharyngeal aphthae healed and inflammatory parameters decreased (WBC 10.98 × 10^9^/L, CRP 26.5 mg/L) so that she could be discharged after 9 days continuing antibiotic treatment with doxycycline. In the follow up examination 24 days after initial presentation the splenic lesions increased in number and in size (Figure 2). Moreover the inflammatory parameters had risen again (WBC 14.19 × 10^9^/L, CRP 175 mg/L). Subsequently, a computed tomography-guided needle biopsy was performed. The patient was restarted on meropenem and metronidazole. Using this therapy the CRP decreased and the white blood cell count normalized again (WBC 9.86 × 10^9^/L, CRP 75.8 mg/L) within 9 days.

**Figure 3:**
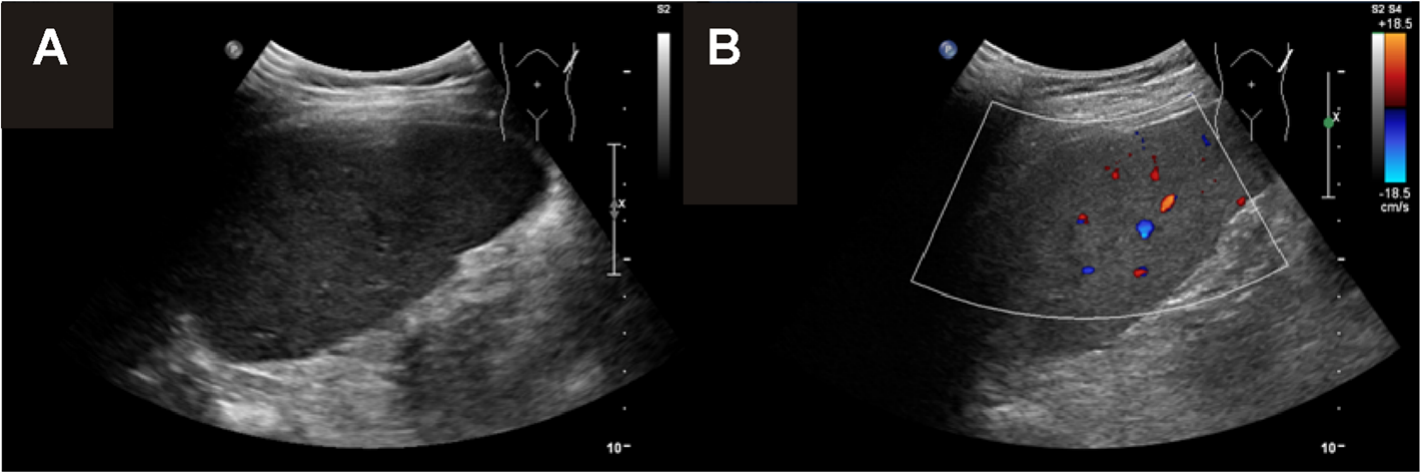
**Ultrasound of the spleen after 6 weeks of glucocorticoid treatment. A**, **B**. Follow up B-mode **(A)** and duplex **(B)** ultrasound of the spleen show a homogenous texture and normal size.

Microscopic analysis of the abscess aspirate showed mainly granulocytes and cell detritus, but no bacteria, fungi or parasites. Repeated blood cultures as well as cultures and 18S and 16S RNA PCR from the splenic biopsy were negative. To exclude concomitant inflammatory bowel disease the patient underwent colonoscopy. On macroscopic evaluation the ileum and colon appeared normal, histologic evaluation of the biopsies taken showed mild chronic inflammation, but no characteristic signs of an inflammatory bowel disease. Antineutrophil cytoplasmic antibodies (ANCA) and anti-*Saccharomyces cerevisiae* antibodies (ASCA) were negative. An immunosuppressive therapy with prednisone 2 mg/kg was started while at first continuing with metronidazole. After initiation of the immunosuppressive treatment the splenic lesions decreased in size for the first time and were no longer detectable after 6 weeks (Figure 3). The CRP normalized completely. The glucocorticoid dose was then slowly tapered and stopped after 11 weeks. During follow up of 1 year she still is in clinical and radiological remission. Autoimmune thyreoidits was still subclinical by that time.

## Discussion

Aseptic abscesses represent a new disease entity that was only recently recognized [4,5]. The spleen is the most frequently involved organ, but aseptic abscesses can also be found in abdominal lymph nodes, liver, lung, pancreas or the brain. Moreover, they can occur uni- or multifocal. Their aetiology is idiopathic or can be associated with other conditions such as relapsing polychondritis or inflammatory bowel disease, especially Crohn’s disease. The bowel inflammation can occur concomitantly, manifest prior or after the appearance of the abscesses [4,5]. Therefore, it is still possible that our patient may develop inflammatory bowel disease in the future. However, during the follow-up she has not developed any other gastrointestinal symptoms.

In the paediatric population aseptic abscesses are very rare with only six reported patients younger than 18 years at diagnosis (age range 6 to 17) [4,6,7]. The clinical presentation seems to be similar to those in adults with fever, increased ESR and elevated white blood cell count. In four of the six patients the abscesses were primarily found in the spleen, in one patient only the lung and in another one the site of involvement was not mentioned. Two thirds of patients were reported to have a concomitant disease such as Crohn’s disease, pyoderma gangrenosum and/or relapsing polychondritis. To date, there are no reports of aseptic abscesses in children under the age of 5 years.

To our knowledge, our case is the first patient described with aseptic splenic abscesses in the absence of an overt accompanying clinical disorder, in whom the antibiotic regimen including meropenem and metronidazole improved the patient’s clinical condition, led to the healing of the aphthous ulcers of the throat and decreased systemic inflammatory markers. After stopping this antibiotic therapy inflammatory markers rose again, and could be decreased repeatedly after reintroduction of the same regimen. However, the antibiotic treatment did not have any beneficial effect on the number and size of the splenic abscesses. Of note, in one case of Crohn’s disease with multiple aseptic liver abscesses antibiotic therapy with meropenem and metronidazole also improved the patient’s clinical condition and inflammatory parameters [8]. One possible explanation for this effect could be gram-negative anaerobic bacteria playing an aetiological factor. The effects of the antibiotic treatment could be exerted by the reduction of the luminal bacterial content in the gut that somehow triggers the inflammatory processes like in inflammatory bowel disease. This mode of action would fit into the hypothesis of gut derived chemokines that maintain a persistent local (splenic) inflammatory neutrophil infiltrate. Additionally, direct immunomodulatory effects of metronidazole may play a role [9-11].

Our observations in the presented case are in accordance with previously published data where the diagnosis of aseptic abscesses relies on a combination of typical clinical, laboratory and radiological findings and exclusion of infectious diseases and other causes [5]. Involvement of the pharynx as in our case has once been described in a patient with aseptic abscesses [12]. Of note, autoimmune thyreoiditis has so far not been described as a concomitant autoimmune mediated disease in patients with aseptic abscesses. Improvement and finally complete resolution of the splenic lesions after the patient receives an immunosuppressive therapy is another characteristic feature for aseptic abscesses, although the relapse rate is high with 32 to 66% when the immunosuppressive therapy is stopped [4].

## Conclusions

Aseptic splenic abscesses should prompt the clinician to initiate a work-up of a potential underlying condition. Anaerobic bacteria may be an aetiological factor and metronidazole may have direct immunomodulatory effects to improve signs and symptoms.

## Consent

Written informed consent was obtained from the parent of the patient for publication of this case report and any accompanying images. A copy of the written consent is available for review by the Editor-in-Chief of this journal.

## Competing interests

The authors have declared that they have no competing interests.

## Authors’ contributions

AJJ, RA, HS and TT prepared the manuscript. MS and KWN provided magnetic resonance images. KPB provides the microbiological results. AJJ, RA, HS and TT cared for the patient and provided advice on the clinical aspects of the case report. All authors read and approved the final version of the manuscript.

## Pre-publication history

The pre-publication history for this paper can be accessed here:

http://www.biomedcentral.com/1471-230X/14/20/prepub
